# Supplementary material for: Dietary Flavonoid Intake and Anemia Risk in Children and Adolescents: Insights from National Health and Nutrition Examination Survey
Source: Antioxidants (Basel). 2025 Mar 27;14(4):395. doi: 10.3390/antiox14040395 (PMC12024375; doi:10.3390/antiox14040395)
Supplement: Supplementary file 1 [file antioxidants-14-00395-s001.zip › antioxidants-3490647-supplementary.pdf]

**Supplementary Table S1.** Distributions and concentrations of dietary flavonoid intakes (mg/day) among children and adolesents in NHANES 2007–2010 and 2017–2018.

| Flavonoid class | Mean | 5th | 25th | 50th | 75th | 95th |
|---|---|---|---|---|---|---|
| ***Isoflavones*** | | | | | | |
| Daidzein | 0.421 | 0.000 | 0.000 | 0.000 | 0.015 | 1.052 |
| Genistein | 0.564 | 0.000 | 0.000 | 0.005 | 0.035 | 1.360 |
| Glycitein | 0.072 | 0.000 | 0.000 | 0.000 | 0.000 | 0.195 |
| Total Isoflavones | 1.058 | 0.000 | 0.000 | 0.005 | 0.006 | 2.706 |
| ***Anthocyanidins*** | | | | | | |
| Cyanidin | 1.519 | 0.000 | 0.055 | 0.515 | 1.650 | 4.342 |
| Delphinidin | 0.781 | 0.000 | 0.000 | 0.020 | 0.46 | 3.285 |
| Malvidin | 2.926 | 0.000 | 0.000 | 0.005 | 1.583 | 16.283 |
| Pelargonidin | 1.371 | 0.000 | 0.000 | 0.005 | 0.285 | 9.026 |
| Peonidin | 0.738 | 0.000 | 0.000 | 0.007 | 0.455 | 2.250 |
| Petunidin | 0.588 | 0.000 | 0.000 | 0.015 | 0.320 | 2.410 |
| Total anthocyanidins | 7.924 | 0.000 | 0.160 | 0.178 | 7.578 | 35.473 |
| ***Flavan-3-ols*** | | | | | | |
| (-)-Epicatechin* | 8.109 | 0.015 | 1.823 | 5.925 | 11.690 | 23.890 |
| (-)-Epicatechin 3-gallate* | 2.611 | 0.000 | 0.000 | 0.010 | 0.110 | 16.521 |
| (-)-Epigallocatechin* | 4.19 | 0.000 | 0.015 | 0.180 | 0.625 | 25.100 |
| (-)-Epigallocatechin 3-gallate* | 6.862 | 0.000 | 0.000 | 0.045 | 0.280 | 44.823 |
| (+)-Catechin* | 4.733 | 0.075 | 1.665 | 3.725 | 6.565 | 12.645 |
| (+)-Gallocatechin* | 0.408 | 0.000 | 0.000 | 0.000 | 0.015 | 2.520 |
| Theaflavin | 0.396 | 0.000 | 0.000 | 0.000 | 0.000 | 2.632 |
| Theaflavin-3,3'-digallate | 0.436 | 0.000 | 0.000 | 0.000 | 0.000 | 2.925 |
| Theaflavin-3'-gallate | 0.370 | 0.000 | 0.000 | 0.000 | 0.000 | 2.490 |
| Theaflavin-3-gallate | 0.314 | 0.000 | 0.000 | 0.000 | 0.000 | 2.101 |
| Thearubigins | 23.862 | 0.000 | 0.000 | 0.000 | 0.000 | 152.981 |
| Total Flavan-3-ols | 52.220 | 0.209 | 4.745 | 11.970 | 26.515 | 269.784 |
| ***Flavanones*** | | | | | | |
| Eriodictyol | 0.165 | 0.000 | 0.000 | 0.000 | 0.090 | 0.910 |
| Hesperetin | 10.416 | 0.000 | 0.000 | 1.015 | 14.638 | 45.736 |
| Naringenin | 2.917 | 0.00 | 0.010 | 0.275 | 3.230 | 14.142 |
| Total Flavanones | 13.498 | 0.000 | 0.015 | 1.430 | 19.295 | 59.607 |
| ***Flavones*** | | | | | | |
| Apigenin | 0.064 | 0.000 | 0.005 | 0.015 | 0.060 | 0.275 |
| Luteolin | 0.353 | 0.000 | 0.045 | 0.170 | 0.430 | 1.290 |
| Total Flavones | 0.418 | 0.000 | 0.070 | 0.220 | 0.520 | 1.482 |
| ***Flavonols*** | | | | | | |
| Isorhamnetin | 0.364 | 0.000 | 0.005 | 0.155 | 0.460 | 1.417 |
| Kaempferol | 1.585 | 0.040 | 0.335 | 0.850 | 1.830 | 5.633 |
| Myricetin | 0.526 | 0.010 | 0.085 | 0.205 | 0.525 | 2.187 |
| Quercetin | 6.099 | 0.935 | 2.638 | 4.785 | 7.955 | 15.968 |
| Total Flavonols | 8.575 | 1.294 | 3.656 | 6.395 | 10.730 | 23.416 |
| ***Total sum of all 29 flavonoids*** | 83.691 | 5.233 | 19.203 | 40.590 | 83.523 | 315.810 |

5th, 5th percentile; 25th, 25th percentile; 50th, 50th percentile; 75th, 75th percentile; 95th, 95th percentile.
